# Targeting the equilibrative nucleoside transporter ENT1 in Huntington disease

**DOI:** 10.18632/oncotarget.15111

**Published:** 2017-02-06

**Authors:** Xavier Guitart, Yijuang Chern, Sergi Ferré

**Affiliations:** Integrative Neurobiology Section, National Institute on Drug Abuse, Intramural Research Program, National Institutes of Health, Baltimore, MD, USA

**Keywords:** Huntington disease, adenosine, ENT1, adenosine A2A receptor, Neuroscience

Huntington disease (HD) is a neurodegenerative disorder phenotypically characterized by progressive chorea, cognitive impairments and emotional disturbances. The crucial pathological hallmark of the disease is the atrophy of the striatum, with a remarkable loss of projecting medium spiny neurons.

Among many disturbances in the homeostasis of the striatum reported in HD, it has been suggested that impairment in adenosine neurotransmission plays an important role in the neurodegenerative process and therefore the progression of the disease [1]. Epidemiological data implicate adenosine receptors of the A_2A_ subtype. Thus, several polymorphisms in the A_2A_ receptor gene have been associated to a reduced age of onset of HD [2], and usual intake of the non-selective adenosine receptor caffeine was also significantly associated with a reduced age of onset of the disease by about 4 years [3]. We have previously shown, using a transgenic rat model expressing a fragment of the *HTT* (huntingtin) gene with 51 CAG repeats (Tg51 rats) that an A_2A_ receptor antagonist does not induce any increase in locomotor activity, first suggesting a possible alteration in the receptor density or function [4]. More recently, we have described that an A_2A_ receptor agonist produced very similar effects reducing locomotor activity either in the transgenic rats or the wild-type littermate controls and radioligang-binding experiments showed no differences either in the binding properties of striatal A_2A_ receptors [4]. All these experimental results brought us to consider the possibility of a low adenosine tone in this HD animal model. By means of *in vivo* microdialysis experiments we did observe lower levels of adenosine in the striatum of either heterozygous and homozygous Tg51 rats at 12 month of age. The results were reproduced in a more recently introduced animal model of HD, the zQ175 knock-in mouse, which expresses the entire HTT carrying 188 CAG repeats. Basal levels of striatal adenosine were measured by *in vivo* microdialysis in 12 month-old zQ175 knock-in mice and found to be significantly decreased as compared to the levels in the wild-type littermates, although the levels of striatal A_2A_ receptors were also substantially reduced in the knock-in mice [5]. The reduced tone of adenosine drove us to investigate the possible changes in a major player in the process of adenosine reuptake: the equilibrative nucleoside transporter ENT1, a protein mainly expressed by the astrocytes in the brain.

Adenosine in the brain may be either released or may be the product of degradation of ATP. Adenosine is mainly removed from the extracellular space by uptake through ENT1. We found that ENT1 was up-regulated in the striatum of the zQ175 mice, which clearly points out to this protein as a responsible for the low adenosinergic tone [5]. Importantly, the expression of the ENT1 transcript *(SLC29A1)* was also significantly increased in human postmortem prefrontal cortex from HD patients with a grade 2 Vonsattel neuropathological severity score and not in more severe stages (Vonsattel severity score 3 and 4) [6]. We also found using a differential coexpression analysis that *SLC29A1* serves as a hub of HD-induced gene dysregulation, with ENT1 showing gained of correlations with other genes in the HD group as compared to controls.

Taking together, all these data show that ENT1 and extracellular adenosine levels can be used as biomarkers of initial stages of neurodegeneration in HD and that ENT1 might represent a therapeutic target, while the use of adenosine receptor agonists is not recommended given the unwanted peripheric effects. On the other hand, ENT1 levels could be measured in the periphery, where they have been described in erythrocytes and leucocytes. The use of this approach presents though a caveat because it is not known if the up-regulation of ENT1 in the brain parallels with an up-regulation in peripheral cells.

Given that inhibitors of ENT1 such as dipyridamole, ticagrelor or dilazep have already been used to treat different pathological conditions related to vascular relaxation and platelet aggregation, or the NSAID sulindac sulfide for its anti-inflammatory effects, it would be interesting to use these compounds in clinical studies with people affected by HD in order to see if it is possible to partially reduce the progression of the disease or to delay in a significant way the age of onset.

This work was funded by the IRP-NIDA

## References

[1] Lee CF (2014). Int Rev Neurobiol.

[2] Dhaenens CM (2009). Neurobiol Dis.

[3] Simonin C (2013). Neurobiol Dis.

[4] Orrú M (2011). Exp Neurol.

[5] Guitart X (2016). Neurobiol Dis.

[6] Vonsattel JP (1985). J Neuropathol Exp Neurol.

